# No clear association between time to return‐to‐play and first additional injury in European professional football players after ACL injury: A media‐based study

**DOI:** 10.1002/jeo2.70934

**Published:** 2026-10-03

**Authors:** Andrea Marchetti, Michelangelo Scordo, Lorenzo Scarpi, Massimiliano Colotti, Belinda Trobec, Raffaele Sabot, Manuela Deodato, Gianluca Canton, Luigi Murena, Ramana Piussi

**Affiliations:** ^1^ Orthopaedics and Traumatology Unit, Department of Medical, Surgical and Health Sciences Cattinara Hospital—ASUGI, Trieste University Trieste Italy; ^2^ Department of Medicine, Surgery and Health Sciences School of Physiotherapy, University of Trieste Trieste Italy; ^3^ Sahlgrenska Sports Medicine Center Gothenburg Sweden; ^4^ Unit of Physiotherapy, Department of Health and Rehabilitation Institute of Neuroscience and Physiology, Sahlgrenska Academy, University of Gothenburg Gothenburg Sweden

**Keywords:** injury surveillance, sports epidemiology, survival analysis

## Abstract

**Purpose:**

The primary aim of this media‐based study was to investigate whether time from anterior cruciate ligament (ACL) injury to return to play (RTP) was associated with the hazard of a first additional injury over calendar time among male professional football players in the five major European leagues. Secondary aims were to examine associations with a first additional injury within 365 days after RTP and with the number of official competitive matches missed because of the first additional injury.

**Methods:**

Male professional football players who sustained an ACL injury, underwent ACL reconstruction and returned to official competition were identified from publicly available data. RTP was defined as the first official competitive match after ACL injury. Cox proportional hazards regression assessed associations over the full follow‐up and within 365 days, adjusted for age and player position. Sensitivity analysis examined predefined RTP categories; negative binomial regression assessed the association between time to RTP and the number of official competitive matches missed because of the first additional injury.

**Results:**

Among 278 players, mean time to RTP was 242.3 ± 99.5 days and 201 (72.3%) sustained a first additional injury. No clear evidence of an association was observed between time to RTP and the hazard of a first additional injury over the full follow‐up or within 365 days. Similarly, no clear evidence of an association was observed in the categorical sensitivity analysis or between time to RTP and the number of matches missed because of the first additional injury.

**Conclusions:**

No clear evidence of an association was observed between time from ACL injury to RTP and the hazard of a first additional injury either over the full follow‐up period or within 365 days after RTP. Similarly, no clear evidence of an association was observed between time to RTP and the number of official competitive matches missed because of the first additional injury.

**Level of Evidence:**

Level IV.

## Graphical abstract

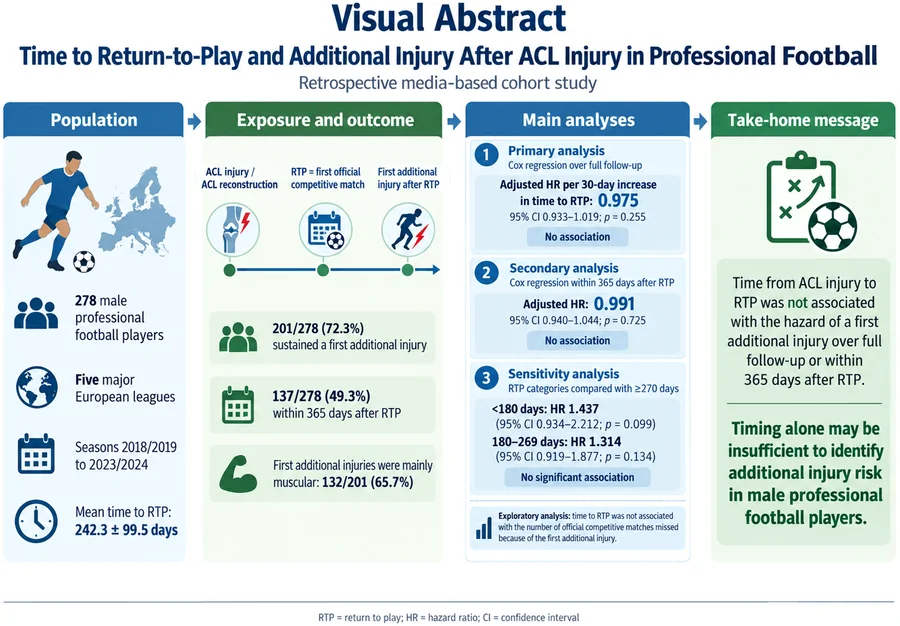

**Abbreviations**
ACL: anterior cruciate ligament
ACLR: anterior cruciate ligament reconstruction
CI: confidence interval
HR: hazard ratio
IQR: interquartile range
OR: odds ratio
RTP: return to play
RTS: return to sport
STROBE: Strengthening the Reporting of Observational Studies in Epidemiology

## INTRODUCTION

Anterior cruciate ligament (ACL) rupture is a serious and potentially career‐limiting injury for professional athletes, particularly in athletes involved in sports that include jumping, hard pivoting and cutting movements [23, 29, 30]. Professional and elite athletes who undergo ACL reconstruction generally have higher return‐to‐play (RTP) rates than non‐professional athletes [7, 9]. Rates of RTP in professional athletes treated with ACL reconstruction range from 77% to 100% [3, 13, 21], with 83% of elite athletes successfully returning to their pre‐injury level of sport [25]. Specifically, among European professional football players, RTP rates after anterior cruciate ligament reconstruction (ACLR) are high and range between 80% and 98% [6, 16]. In elite professional footballers, RTP after ACLR generally occurs within the first year, with players returning to competitive match play after approximately 7 months [32].

Despite the high percentage of European professional football players who successfully RTP after ACLR, the post‐RTP period remains a critical phase. As professional football players RTP after ACLR, the risk of a subsequent knee injury is high, with 50%–80% of subsequent injuries occurring within the first 4 weeks of RTP [16, 34]. Furthermore, RTP after ACLR for professional footballers is followed by an increase in hamstring and quadriceps muscle injuries during the two years following RTP [10]. These findings refer to knee‐specific reinjury and muscle injuries, respectively. Evidence regarding broader additional injuries after RTP, irrespective of anatomical location or injury type, remains limited.

Time to RTP has been reported not to differ between professional athletes who suffered a second ACL injury and those who did not [28]; however, studies that investigate the occurrence of additional injuries (e.g., muscle, tendon or overuse injuries) after RTP and the relationship between time to RTP and these injuries in elite football players remain limited. Contemporary approaches to RTP after ACLR emphasize that timing should be considered together with functional, psychological and sport‐specific factors rather than used as an isolated criterion [1, 15, 19, 33]. Available evidence does not clarify whether the time interval from ACL injury and reconstruction to RTP is associated with a first additional injury or whether any association between time to RTP and an additional injury exists within the first year after RTP. Whether time to RTP is associated with the number of matches missed because of a first additional injury also remains unclear.

Therefore, the primary aim of this media‐based study was to investigate whether time from ACL injury to RTP was associated with the hazard of a first additional injury over calendar time among male professional football players in the five major European leagues. Secondary aims were to examine whether time from ACL injury to RTP was associated with a first additional injury within 365 days after RTP and with the number of official competitive matches missed because of the first additional injury, used as a proxy for injury severity. The occurrence and characteristics of additional injuries were also described.

## METHODS

This retrospective media‐based cohort study primarily investigated whether time from ACL injury to RTP was associated with the hazard of a first additional injury over calendar time among male professional football players from the five major European leagues (Premier League, La Liga, Serie A, Bundesliga and Ligue 1) from the beginning of the 2018/2019 season to the date of data extraction: 1 December 2025. Secondary analyses examined the association between time to RTP and a first additional injury within the first year (365 days) after RTP, as well as the association between time to RTP and the time‐loss of the first additional injury. This study was reported according to the Strengthening the Reporting of Observational Studies in Epidemiology (STROBE) statement [31].

### Included players

Players were eligible if they were under contract with a club competing in one of the five major European leagues at the time of ACL injury and subsequently returned to an official competitive match. Players were excluded if the ACL injury occurred prior to joining a club in one of the included leagues, if they did not RTP following ACL injury, if they retired or lost their contract before RTP, or if the index ACL injury was not a primary ACL rupture.

### Outcomes

#### Definitions

In this study, RTP was defined as participation in the first official competitive match after ACL injury. Time to RTP was defined as the interval in days between ACL injury and RTP. An additional injury was defined as any publicly reported injury that occurred after RTP during the defined follow‐up period. The first additional injury after RTP was used for the primary, secondary and severity analyses. Follow‐up started at RTP and ended at the date of the first additional injury, the last date with reliable follow‐up or the date of data extraction: 1 December 2025, whichever occurred first. Reliable follow‐up was defined as the last match played for the team in the included leagues for players who transferred, retired, lost their contract or left one of the included leagues before the end of follow‐up. Data were extracted on 1 December 2025 and included injury and match data up to the date of data extraction.

#### Primary outcome

The primary outcome was the first additional injury over calendar time. Calendar time was defined as the number of days from RTP to the first additional injury or censoring. Players without an additional injury were censored at the date of data extraction, or at the last date with reliable follow‐up, whichever occurred first.

#### Secondary outcome

The secondary outcome was the first additional injury within the first year (365 days) after RTP. Players without an additional injury within 365 days were censored at 365 days, at the date of data extraction of 1 December 2025, or the last date with reliable follow‐up, whichever occurred first. Additional injuries that occurred more than 365 days after RTP were not considered events in the secondary analysis.

#### Matches missed and descriptive time‐loss categories

For the inferential analysis, the number of official competitive matches missed because of the first additional injury was used as a proxy for injury severity. Separately, for descriptive purposes, additional injuries were categorized according to duration of absence as minimal (1–3 days), mild (4–7 days), moderate (8–28 days) or severe (>28 days), when the duration of absence was available. The time‐loss category was recorded as missing when the duration of absence was unavailable. Additional injury type was classified as tendon, ligament, muscle or other [17]. The occurrence, type, time‐loss category and number of matches missed were described for the first and second additional injuries.

### Data collection

Injury data were identified using publicly available data. The identification process was based on methodologies used in previous epidemiological studies on professional athletes [2, 8, 14, 22]. Transfermarkt served as the primary database for identification of eligible players and extraction of study variables. Potentially eligible ACL injury cases were identified across the Premier League, La Liga, Serie A, Bundesliga and Ligue 1 from the 2018/2019 season to the database‐lock date of 1 December 2025. The search was performed by three authors (A.M, L.S. and M.C.). Both investigator (L.S. and M.C.) extracted the data independently and recorded them in a database created using Microsoft Excel, version 16.111.1 (Microsoft Corporation). Discrepancies were resolved by consultation with the first author (A.M.). Duplicate records were identified by comparing player name, date of birth, club, league and ACL injury date. Records referring to the same player and the same index ACL injury episode were merged into a single database entry. When duplicate records contained complementary information, the available data were combined. In case of conflicting information, data from official club communications were prioritized; when official information was unavailable, the value supported by the most consistent and independently corroborated sources was retained.

Data for league, season, club, age at ACL injury, date of RTP, number of matches missed because of the index ACL injury, the occurrence and characteristics of additional injuries after RTP and player position were extracted. The player position was defined as the player's main position when the injury occurred, including goalkeeper, defender, midfielder and forward. Additional‐injury variables included the date of injury, reported diagnosis or injury description, injury type, duration of absence, date of return to competition and number of official competitive matches missed. Data for the first and second additional injuries were extracted separately. Each ACL injury and additional‐injury record was cross‐checked against at least one independent publicly available source. Official club communications, including medical bulletins and injury reports published on club websites, had priority. When official club information was unavailable, reports from established sports news outlets served as corroborating sources [17]. Records without an independent corroborating source and/or information that could not be verified were excluded.

The RTP was verified through the first official competitive match after the ACL injury. Follow‐up records were reviewed from RTP until the first additional injury or the database‐lock date of 1 December 2025. The dates and characteristics of the first and second additional injuries were recorded separately.

Before statistical analysis, the database was reviewed for duplicate entries, impossible date sequences, missing values and inconsistencies between dates and calculated intervals. Quality control was performed by R.P. Any discrepancy identified during quality control was reassessed against the original sources and resolved through discussion.

### Statistics

Statistical analyses were performed with the Statistical Product and Service Solutions (IBM Corp., Released 2017; IBM SPSS Statistics for Windows, Version 25.0; IBM Corp.). Descriptive statistics comprising age, playing position, matches missed due to ACL injury and time to RTP were displayed for all included players. Further, the proportion of players who suffered one or two additional injuries, the type and severity of additional injuries and the matches missed due to additional injuries were reported. Continuous variables were presented as mean ± standard deviation or median (interquartile range [IQR]), as appropriate based on data distribution. Categorical variables were presented as frequencies and percentages.

Four analyses were performed: one analysis of the primary outcome, one of the secondary outcome, a sensitivity analysis and a severity analysis. For the primary and secondary aims of the study, Cox proportional hazards regression models were used to examine whether time from ACL injury to RTP was associated with the hazard of a first additional injury over calendar time (primary analysis) or within 365 days after RTP (secondary analysis). The time scale was the number of days from RTP to the first additional injury or censoring. In the primary analysis, players without a first additional injury were censored at the last date with reliable follow‐up or at the database‐lock date of 1 December 2025, whichever occurred first. In the secondary analysis, players were censored at 365 days after RTP, the last date with reliable follow‐up or the database‐lock date of 1 December 2025, whichever occurred first. Time to RTP was scaled per 30‐day increase to provide clinically meaningful hazard ratios (HRs). The proportional hazards assumption for time to RTP was assessed by including an interaction between time to RTP and the natural logarithm of follow‐up time in each Cox model. No evidence of violation of the proportional hazards assumption was observed. The models were presented unadjusted and adjusted for age at index ACL injury and player position (goalkeeper, defender, midfielder, striker). Age and player position were selected as clinically relevant player‐level covariates because both may plausibly be associated with the timing of RTP and post‐RTP injury risk. The HRs with 95% confidence intervals (CIs) were reported. For the sensitivity analysis, the primary Cox regression was repeated with time from ACL injury to RTP categorized as <180, 180–269 and ≥270 days. The ≥270‐day category served as the reference. For the severity analysis, this study aimed to assess whether time to RTP was associated with the severity of additional injuries, defined as the number of official competitive matches missed due to the additional injury. In this analysis, only the first additional injury was considered. The analysis included only players who sustained a first additional injury and for whom the number of matches missed was available. Negative binomial regression was used because the number of matches missed represented count data with a right‐skewed and overdispersed distribution. Time to RTP was modelled per 30‐day increase. The analysis was performed unadjusted and adjusted for age at index ACL injury and player position. Exponentiated regression coefficients (Exp[B]) with 95% CIs were reported and represent the ratio of expected numbers of matches missed per 30‐day increase in time to RTP. The significance level was set at *α* = 0.05.

## RESULTS

A total of 278 male football players were included in the present study. The study flowchart is shown in Figure 1, and the demographic characteristics of the included players are reported in Table 1. Included players were, on average, 25.5 ± 4.1 (median 25 [IQR 22–29]) years old at the time of index ACL injury, and defender was the most common playing position (118/278, 42.4%). Players missed a mean of 33.2 ± 14.9 matches due to the index ACL injury, and the mean time to RTP was 242.3 ± 99.5 days. After RTP, 201/278 players (72.3%) sustained at least one additional injury: the first additional injury was most commonly muscular (132/201, 65.7%) or ligamentous (27/201, 13.4%), while tendon injuries were rare (4/201, 2.0%) and 38/201 (18.9%) were classified as other. Regarding the time‐loss category of the first additional injury, 4/201 (2.0%) were minimal, 20/201 (10.0%) were mild, 76/201 (37.8%) were moderate, and 101/201 (50.2%) were severe. These additional injuries resulted in 8.7 ± 13 matches missed on average. A second additional injury occurred in 142/278 players (51.1%): the second additional injuries were predominantly muscular (92/142, 64.8%), followed by ligament injuries (19/142, 13.4%), tendon injuries (6/142, 4.2%) and other injuries (25/142, 17.6%). The time‐loss category of the second additional injury was minimal in 1/142 (0.7%) cases, mild in 16/142 (11.3%) cases, moderate in 62/142 (43.7%) cases and severe in 63/142 (44.4%) cases, with 8 ± 10.7 matches missed on average. Overall, 25 football players (9%) sustained an ACL graft failure during follow‐up.

**Figure 1:**
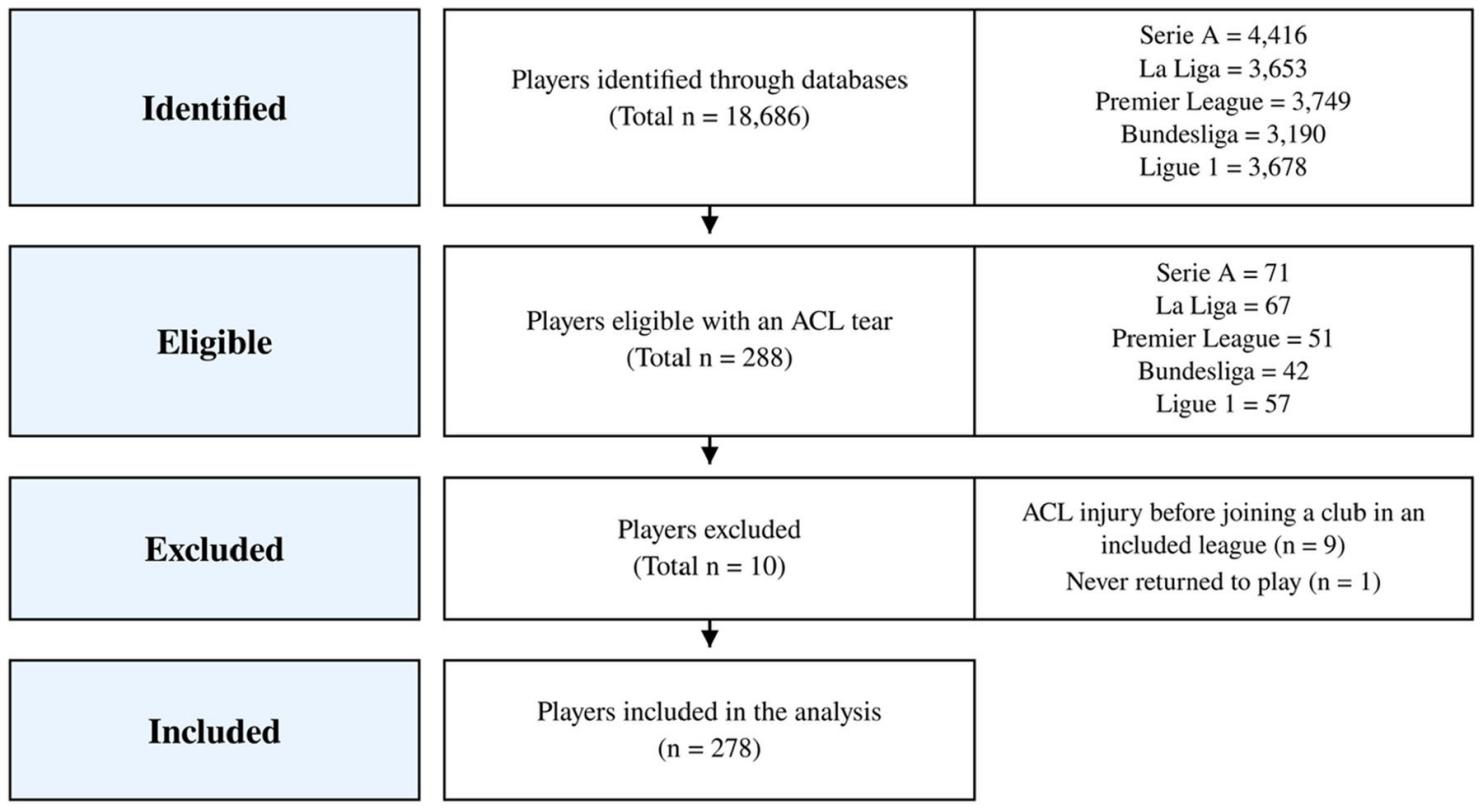
Flow diagram of the study. ACL, anterior cruciate ligament; *n*, number.

**Table 1:** Demographic variables for included players.

Included players, *n*	278
Age at index ACL injury, years, mean ± SD; Median (IQR)	25.5 ± 4.1 25 (22–29)
Player position, *n* (%)	
Goalkeeper	14 (5%)
Defender	118 (42.4%)
Midfielder	60 (21.6%)
Striker	86 (30.9%)
Matches missed due to index ACL injury, mean ± SD	33.2 ± 14.9
Time to RTP (days) from index ACL injury, mean ± SD	242.3 ± 99.5
Players who suffered one additional injury after RTP, *n* (%)	201 (72.3%)
Time between RTP and first additional injury, days, mean ± SD; Median (IQR)	326.4 ± 342 190 (73–487)
Type of first additional injury, *n* (%)	
Tendon	4 (2%)
Ligament	27 (13.4%)
Muscular	132 (65.7%)
Other	38 (18.9%)
Time‐loss category of first additional injury, *n* (%)	
Minimal	4 (2%)
Mild	20 (10%)
Moderate	76 (37.8%)
Severe	101 (50.2%)
Matches missed due to the first additional injury, mean ± SD	8.7 ± 13
Players who suffered two additional injuries after RTP, *n* (%)	142 (51.1%)
Type of second additional injury, *n* (%)	
Tendon	6 (4.2%)
Ligament	19 (13.4%)
Muscular	92 (64.8%)
Other	25 (17.6%)
Time‐loss category of second additional injury, *n* (%)	
Minimal	1 (0.7%)
Mild	16 (11.3%)
Moderate	62 (43.7%)
Severe	63 (44.4%)
Matches missed due to the second additional injury, mean ± SD	8 ± 10.7
ACL graft failures, *n* (%)	25 (9%)

Abbreviations: ACL, anterior cruciate ligament; IQR, interquartile range; *n*, number; RTP, return to play; SD, standard deviation.

Median follow‐up duration was 340 days (IQR 114–796) in the primary analysis and 340 days (IQR 114–365) in the secondary 365‐day analysis. In the primary analysis, 201 players sustained a first additional injury, and 77 were censored at the database‐lock date; no player was censored earlier because of loss of reliable follow‐up. In the secondary analysis, 137 players sustained a first additional injury and 141 were censored; 133 at the prespecified 365‐day limit and 8 at the database‐lock date before reaching 365 days.

### Primary outcome: First additional injury over calendar time

For the primary outcome, no clear evidence of an association was observed between time from ACL injury to RTP and the hazard of a first additional injury over calendar time. In the unadjusted model, the HR per 30‐day increase in time to RTP was 0.981 (95% CI 0.940–1.024; *p* = 0.385). After adjustment for age at ACL injury and player position, the HR was 0.975 (95% CI 0.933–1.019; *p* = 0.255) (Table 2).

**Table 2:** Results of the primary Cox proportional hazards regression over calendar time.

	Unadjusted		Adjusted^a^	
	HR (95% CI)	*p* value	HR (95% CI)	*p* value
Time to RTP (per 30‐day increase)	0.981 (0.940–1.024)	0.385	0.975 (0.933–1.019)	0.255

Abbreviations: ACL, anterior cruciate ligament; CI, confidence interval; HR, hazard ratio; RTP, return to play.

a Adjusted for age at index ACL injury and player position (goalkeeper, defender, midfielder, striker).

### Secondary outcome: First additional injury within the first year (365 days)

In the first 365 days after RTP, 137 (49.3%) players suffered an additional injury. No clear evidence of an association was observed between time from ACL injury to RTP and the hazard of a first additional injury within 365 days after RTP. In the unadjusted model, the HR per 30‐day increase in time to RTP was 0.993 (95% CI 0.943–1.046; *p* = 0.799). After adjustment for age at ACL injury and player position, the HR was 0.991 (95% CI 0.940–1.044; *p* = 0.725; Table 3).

**Table 3:** Results of the secondary Cox proportional hazards regression over 1 year (365 days).

	Unadjusted		Adjusted^a^	
	HR (95% CI)	*p* value	HR (95% CI)	*p* value
Time to RTP (per 30‐day increase)	0.993 (0.943–1.046)	0.799	0.991 (0.940–1.044)	0.725

Abbreviations: ACL, anterior cruciate ligament; CI, confidence interval; HR, hazard ratio; RTP, return to play.

a Adjusted for age at index ACL injury and player position (goalkeeper, defender, midfielder, striker).

The time‐dependent interaction was not statistically significant in either the primary (*p* = 0.363) or secondary Cox model (*p* = 0.719), indicating no evidence of violation of the proportional hazards assumption for time to RTP.

### Sensitivity analysis: Time from ACL injury to RTP categorized as <180, 180–269 and ≥270 days

In the sensitivity analysis, no clear evidence of an association was observed between RTP category (<180, 180–269 and ≥270 days) and the hazard of a first additional injury over calendar time in either the unadjusted (overall *p* = 0.381) or adjusted model (overall *p* = 0.207). Compared with RTP at ≥270 days, the adjusted HR was 1.437 (95% CI 0.934–2.212; *p* = 0.099) for RTP at <180 days and 1.314 (95% CI 0.919–1.877; *p* = 0.134) for RTP at 180–269 days (Table 4).

**Table 4:** Results of the sensitivity Cox proportional hazards regression with RTP categorized as <180 days, 180–269 days and ≥270 days.

			Unadjusted		Adjusted^a^	
Time to RTP	Categories	Number of events/total	HR (95% CI)	*p* value	HR (95% CI)	*p* value
	<180 days versus ≥270	44/54 versus 44/70	1.335 (0.879–2.028)	0.175	1.437 (0.934–2.212)	0.099
	180–269 days versus ≥270	113/154 versus 44/70	1.206 (0.851–1.709)	0.292	1.314 (0.919–1.877)	0.134

Abbreviations: ACL, anterior cruciate ligament; CI, confidence interval; HR, hazard ratio; RTP, return to play.

a Adjusted for age at index ACL injury and player position (goalkeeper, defender, midfielder, striker).

### Severity analysis: Official competitive matches missed because of the first additional injury

Among the 201 players who sustained a first additional injury, no clear evidence of an association was observed between time to RTP and the number of official competitive matches missed because of the first additional injury. The unadjusted Exp(*B*) per 30‐day increase in time to RTP was 1.037 (95% CI 0.989–1.088; *p* = 0.131), and the adjusted Exp(*B*) was 1.011 (95% CI 0.963–1.062; *p* = 0.650) (Table 5).

**Table 5:** Negative binomial regression of the number of official competitive matches missed because of the first additional injury.

	Unadjusted		Adjusted^a^	
	Exp (*B*) (95% CI)	*p* value	Exp (*B*) (95% CI)	*p* value
Time to RTP (per 30‐day increase)	1.037 (0.989–1.088)	0.131	1.011 (0.963–1.062)	0.650

Abbreviations: ACL, anterior cruciate ligament; CI, confidence interval; Exp(*B*), exponentiated regression coefficient; RTP, return to play.

a Adjusted for age at index ACL injury and player position (goalkeeper, defender, midfielder, striker).

## DISCUSSION

The main findings of this study were that no clear evidence of an association was observed between time from ACL injury to RTP and the hazard of a first additional injury, either over the full calendar‐time follow‐up or within the first 365 days after RTP, among male professional football players. Similarly, the categorical sensitivity analysis provided no clear evidence of an association between RTP before 180 days, between 180 and 269 days or at ≥270 days and the hazard of a first additional injury. Likewise, no clear evidence of an association was observed between time to RTP and the number of official competitive matches missed because of the first additional injury. Taken together, the findings indicate that there is no clear evidence of an association between time to RTP and the hazard or severity of a first additional injury when follow‐up time and censoring were considered. Time to RTP represented time to the first official competitive match after ACL injury and not time to complete functional recovery or return to pre‐injury performance. The similar findings over the full follow‐up period and within 365 days after RTP suggest that the results were not substantially influenced by the inclusion of injuries that occurred only after prolonged follow‐up.

In the literature, it is suggested that allowing return to sport (RTS) after ACLR based only on timing may be associated with a higher risk of subsequent injuries [4]. Furthermore, Grindem et al. [20] reported that each additional month of delayed RTP was associated with a 51% reduction in the rate of secondary knee injury. More recently, Kotsifaki et al. reported that, among male athletes who met discharge criteria after ACLR, time to RTS was not associated with the risk of a new knee or ACL injury [24]. The present findings did not provide clear evidence of a comparable association, either when time to RTP was analysed as a continuous variable or when predefined RTP categories were examined. The difference may partly reflect differences in study populations and outcomes, as Grindem et al. [20] examined secondary knee injuries, whereas the present study included all types of additional injuries among male professional football players. The findings therefore do not indicate that timing is irrelevant but suggest that time to RTP after ACL injury alone may be insufficient to identify the risk of an additional injury in professional football players.

The lack of clear evidence of an association both over the full follow‐up period and within the first 365 days after RTP highlights that factors other than the timing of RTP itself may contribute to additional injury susceptibility. For elite male footballers, the transition from rehabilitation to exposure to competitive matches can represent a step‐change in external and internal loading, as the incidence of injuries in matches exceeds the incidence of injuries sustained in training, and the injury risk appears to be particularly elevated in the first match after RTP [5, 12]. In professional football, calendar congestion has been associated with increased injury rates during matches, while overload during matches, combined with international travel, may increase injury incidence [26]. Match demands also vary based on contextual and technical–tactical factors, which also justifies the variety and incidence of injuries to players of different roles, while exposure time to post‐injury matches in professional players is managed in a personalized way and with gradual return‐to‐performance frameworks [11]. Overall, these factors support the rationale that injury susceptibility is associated with exposure and play context beyond the time elapsed between ACL injury and RTP.

Another important aspect of the data in this study is the predominance of muscle injuries among additional injuries after ACL injury and reconstruction, which represent 65.7% of first injuries and 64.8% of second injuries, respectively. This result is plausible following ACLR, as residual neuromuscular deficits, interlimb force asymmetries, altered muscle biomechanics and reduced load tolerance may persist beyond RTP, thereby increasing susceptibility to muscle injury [18, 27]. Consistently, data from the Union of European Football Associations (UEFA) Elite Club Injury Study showed a higher thigh muscle injury incidence within 2 years after RTP in professional male soccer players after ACLR compared with players without ACL injury history (relative risk 1.6) and compared with the same players in the 2 years preceding the index ACL injury (relative risk 2.0), with a particularly marked increase in quadriceps injuries among players reconstructed with a patellar tendon autograft (relative risk 3.5) [10]. Furthermore, graft harvest–related donor‐site morbidity and associated muscle weakness due to arthrogenic muscle inhibition, together with post‐harvest compensatory adaptations (e.g., biceps femoris compensation for semitendinosus function after hamstring‐tendon autograft), have been proposed as plausible mechanisms that may contribute to an increased susceptibility to thigh muscle injuries after ACLR, although their direct influence on subsequent injury risk remains to be established [10].

No clear evidence of an association was observed between time to RTP and the number of official competitive matches missed because of the first additional injury. This result should be interpreted with caution as the number of missed matches is influenced by multiple factors independent of clinical status (number of players in the squad, schedule, technical‐tactical choices) and may not reflect the true severity of the injury. RTP should be closely related to progressive exposure to the match, functional outcomes, targeted neuromuscular reconditioning, secondary prevention as well as normal biological healing times [5].

### Limitations

This study is limited by its retrospective, media‐based design. Injury identification and grading were derived from publicly available sources and, despite cross‐checking with additional reports, may be subject to misclassification and under‐reporting, particularly for less clearly reported injuries. Key clinical and contextual variables were unavailable (e.g., graft type, concomitant lesions, type of post‐injury rehabilitation, match/training exposure and load), which may confound lack of associations between time to RTP and additional injury outcomes. Importantly, individual match and training exposure after RTP was unavailable. The analyses therefore evaluated injury hazard over calendar time and could not account for differences in the number of matches or playing minutes accumulated before injury or censoring. Moreover, RTP was defined as participation in the first official competitive match and therefore represents a media‐observable milestone rather than full functional recovery, clinical readiness or return to pre‐injury performance. Furthermore, the outcome combined injuries with different anatomical locations and mechanisms, which may have obscured associations specific to individual injury types. The number of matches missed is also an imperfect measure of injury severity because it may be influenced by match schedules, squad selection and clinical or managerial decisions. Potential within‐club clustering was not accounted for in the analyses because club membership was not considered a sufficiently stable proxy for shared rehabilitation or medical management across the study period. Finally, residual confounding remains likely because adjustment was limited to the available variables. The findings may not be generalizable beyond male professional football players who returned to competition in the five major European leagues.

## CONCLUSION

In conclusion, among professional male football players in the five major European leagues, no clear evidence of an association was observed between time from ACL injury to RTP and the hazard of a first additional injury, either over the full calendar‐time follow‐up or within the first 365 days after RTP. Similarly, no clear evidence of an association was observed between time to RTP and the number of official competitive matches missed because of the first additional injury. The timing to RTP appears insufficient to identify additional injury risk after ACL injury and reconstruction, and these findings should be interpreted within the limitations of this retrospective, media‐based cohort.

## AUTHOR CONTRIBUTIONS

All listed authors have contributed substantially to this work: Andrea Marchetti, Michelangelo Scordo and Ramana Piussi were responsible for the conception and design of the study. Andrea Marchetti and Ramana Piussi performed study planning, methodology and editing. Lorenzo Scarpi and Massimiliano Colotti conducted the database searches. Ramana Piussi performed the formal analysis. Andrea Marchetti, Michelangelo Scordo and Ramana Piussi wrote the initial draft of the manuscript, which was subsequently edited and revised by all authors. All authors read and approved the final manuscript.

## FUNDING INFORMATION

The authors have no funding to report.

## CONFLICT OF INTEREST STATEMENT

The authors declare no conflicts of interest.

## ETHICS STATEMENT

The authors have nothing to report.

## Data Availability

Data are available from the corresponding author upon reasonable request.
